# The Usefulness of the Breast Density Assessment Application Used by Breast Radiologists

**DOI:** 10.7759/cureus.62560

**Published:** 2024-06-17

**Authors:** Chiharu Kai, Takako Morita, Ikumi Sato, Akifumi Yoshida, Naoki Kodama, Satoshi Kasai

**Affiliations:** 1 Department of Radiological Technology, Faculty of Medical Technology, Niigata University of Health and Welfare, Niigata, JPN; 2 Department of Health and Welfare, Graduate School, Niigata University of Health and Welfare, Niigata, JPN; 3 Department of Breast Surgery, National Hospital Organization, Nagoya Medical Center, Aichi, JPN; 4 Department of Nursing, Faculty of Nursing, Niigata University of Health and Welfare, Niigata, JPN

**Keywords:** screening of breast cancer, inter-variability, mammary gland content ratio, breast density, mammogram

## Abstract

Breast density determined by breast radiologists and also automatically estimated by applications has been widely investigated. However, no study has yet clarified whether the use of these applications by breast radiologists improves reading efficacy. Therefore, this study aimed to assess the usefulness of applications when used by breast radiologists. A *Breast Density Assessment* application (App) developed by Konica Minolta, Inc. (Tokyo, Japan) was used. Independent and sequential tests were conducted to assess the usefulness of the concurrent- and second-look modes. Fifty and 100 cases were evaluated using sequential and independent tests, respectively. Each dataset was configured based on the evaluation by an expert breast radiologist who developed the Japanese guidelines for breast density. Nine breast radiologists evaluated the mammary gland content ratio and breast density; the inter-observer and expert-to-observer variability were calculated. The time required to complete the experiments was also recorded. The inter-observer variability was significant with the App, as revealed by the independent test. The use of the App significantly improved the agreement between the responses of the observers for the mammary gland content ratio and those of the expert by 6.6% and led to a reduction of 186.9 seconds in the average time required by the observers to evaluate 100 cases. However, the results of the sequential test did not suggest the effectiveness of the App. These findings suggest that the concurrent use of the App improves reading efficiency.

## Introduction

Mammography is the only testing modality that has been proven to reduce mortality due to breast cancer; therefore, it is commonly used for the screening of breast cancer [1,2]. However, breast tissue and abnormal lesions have similar densities in mammography. Therefore, abnormal lesions may be overlooked or missed in patients with large amounts of mammary tissue [3,4].

Breasts can be classified into four categories based on the amount of mammary tissue, which is also known as breast density. The guidelines for evaluating breast density were established by the Japan Central Organization on Quality Assurance of Breast Cancer Screening (QABCS) based on the Breast Imaging Reporting Data System (BI-RADS) atlas [5] developed by the American College of Radiology (ACR) [6]. According to the QABCS guidelines, the mammary gland content ratio is determined by calculating the ratio of the mammary gland area equal to or greater than the density of the pectoralis muscle to the area of the breast where the mammary tissues are thought to be present. Breasts with mammary gland content ratios of <10%, 10% to <50%, 50% to <80%, and ≥80% are defined as *fatty*, *scattered*, *heterogeneously dense*, and *extremely dense*, respectively. *Fatty* and *scattered* breasts are grouped as *fatty breasts*, whereas *heterogeneously dense* and *extremely dense* are grouped as *dense breasts*. *Dense breasts*, characterized by a larger area of mammary tissue, have a greater chance of overlapping with abnormal lesions. This overlap poses challenges in detecting abnormal lesions, increasing the risk of missed lesions [3,4], and subsequently, elevating the risk of breast cancer [7-9]. The U.S. Food and Drug Administration mandated informing patients of their breast density in March 2023 [10]; thus, it is essential to evaluate breast density during breast cancer screening and daily medical care and promote an understanding of breast density.

The evaluation of breast density is subjective, relying on the biases of the breast radiologist; thus, inter-radiologist variability remains a source of concern [11-13]. The inter-radiologist agreement rate for breast density tends to be lower in mammograms with mammary gland content ratios close to the thresholds. Redondo et al. [14] reported that inter-radiologist variability was particularly noticeable between the threshold line of *scattered* and *heterogeneously dense* breasts, as well as *fatty* and *dense* breasts, which raises concerns regarding the accuracy of breast density evaluation. Furthermore, the requirements for breast density notification may increase the time required to evaluate the breast density, thereby increasing the total reading time for radiologists.

Several applications have been developed to automatically classify breast density [15,16]. These systems provide objective indicators to assist breast radiologists in determining breast density. Several studies have been conducted on applications that automatically estimate breast density, comparing the results of such applications against those of visual assessments by breast radiologists [17-22]. However, no study has assessed the usefulness of these applications when used by breast radiologists. These applications are designed to reduce potential problems by improving inter-radiologist variability in breast density evaluations and reducing the reading time. Moreover, it is also important to optimize application usage to maximize the abovementioned effects. Therefore, the present study aimed to examine the usefulness of two-dimensional breast density analysis performed by breast radiologists who routinely read mammograms. Thus, an observer experiment was conducted to assess the degree of inter-radiologist variability in breast density evaluations and reading time.

## Materials and methods

The observer experiment was approved by the Institutional Review Board of the Niigata University of Health and Welfare (Approval No. 18733-210903). The study was performed per the ethical standards laid down in the 1964 Declaration of Helsinki. The detailed methods of experimenting are provided in the following sections.

Observers

Nine breast radiologists (seven women and two men) participated in this study as observers. Three breast radiologists had <5 years of experience reading mammograms, three breast radiologists had 5-15 years of experience, and three breast radiologists had ≥15 years of experience (mean experience: 12.0 ± 9.3 years). We explained the experiment in detail to the observers and obtained their informed consent for participation in this study.

Evaluation experiments and training methods

**Figure 1:**
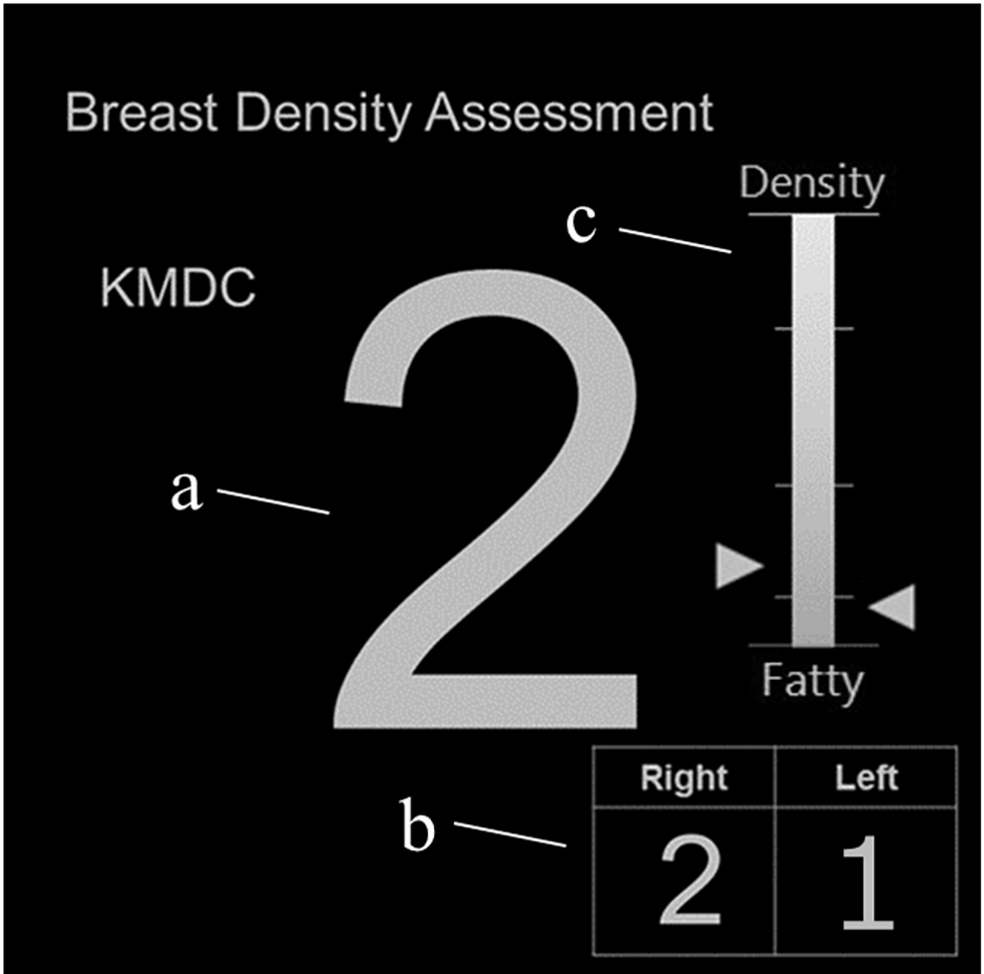
Example of the display of the App. (a) Display of breast density classification results: 1, fatty; 2, scattered; 3, heterogeneously dense; 4, extremely dense. (b) Display of breast density classification results for the right and left breasts. The larger value in (b) is shown in (a). (c) Bar display of the mammary gland content ratios for the right and left breasts.

The *Breast Density Assessment* application (App) manufactured by Konica Minolta Inc. (Tokyo, Japan) was used in this study [23]. The App completes the evaluation via three processes: a process for extracting the area where the mammary tissues are thought to be present upon the reading mammogram after image processing, a process for extracting the areas where mammary glands are present, and a two-dimensional density-based thresholding process for calculating the mammary gland content ratio. QABCS recommends visually determining breast density by reading mammograms, and the App is consistent with these guidelines. A diagram of the App with a representative image of a case is shown in Figure 1. The value in the central panel shows the result of breast density classification per case, and values 1, 2, 3, and 4 depict *fatty*, *scattered*, *heterogeneously dense*, and *extremely dense* classifications, respectively. The results of breast density classification for the right and left breasts are shown in the lower right panel. The larger value is presented as the central figure if the results for the left and right breasts differ. The mammary gland content ratio of the left and right breasts is indicated by a bar, with the upper portion indicating a higher mammary gland content ratio. The bottom and the top of the bar indicate 0% and 100% mammary gland content ratios, respectively. The usefulness of the App when used by radiographers was reported previously [24].

**Figure 2:**
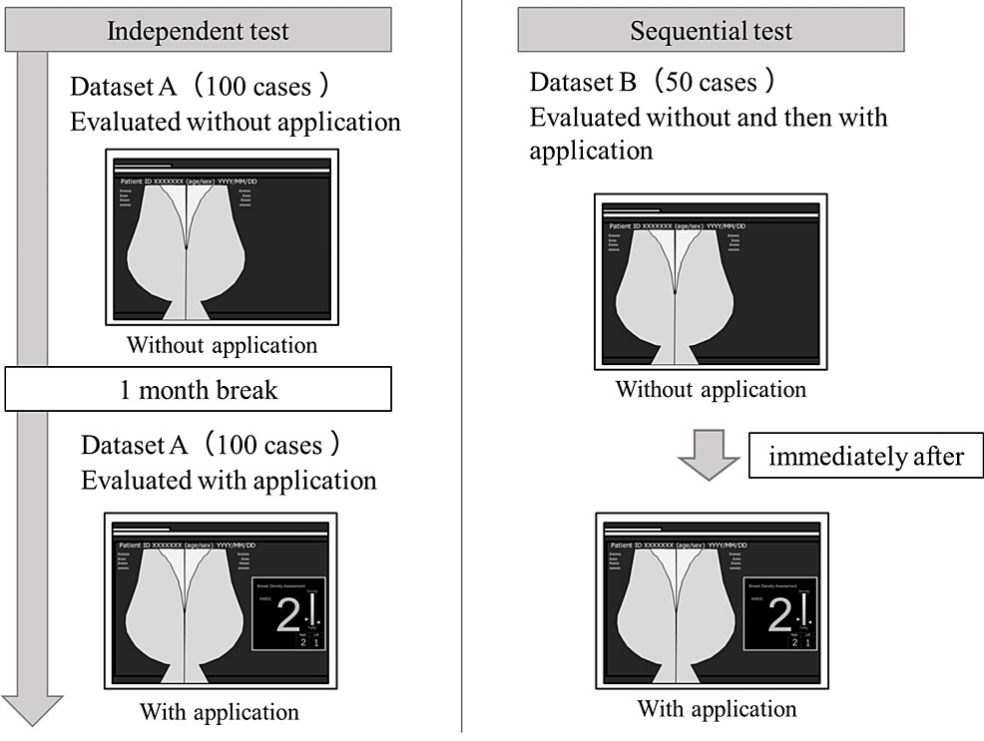
Schematic diagram of the observer experiments (independent test and sequential test). A washout period was set up to ensure that the cases used in the independent test had not been memorized. A sequential test was inserted between the independent tests, with an interim rest period of approximately one month. The independent test in the case without the App and the sequential test were conducted on the same day.

The App has two possible modes: the concurrent look and the second look. The App is presented simultaneously with the image in the concurrent-look mode, and the observer subsequently reads the image. In contrast, the App is presented immediately after the observer reads the image alone in the second-look mode, and the observer subsequently reads the image again using the results of the App. An independent test was conducted to assess the effectiveness of the concurrent-look mode, whereas a sequential test was conducted to assess the effectiveness of the second-look mode. The independent test assesses and compares the reading accuracy when observers read the images without the use of the App versus when the App is presented at the same time as the images. The sequential test assesses and compares reading accuracy when observers read images without the use of the App versus when the App is presented immediately after reading the images and the observers read the images again. Figure 2 presents a schematic diagram of the two experiments. The independent test comprised two observer experiments, including 100 cases, that were conducted with and without the App. The observers first evaluated breast density without the App. After approximately one month, to avoid any impact of the learning effect, the observers evaluated the breast density of the same 100 cases using the App. The order of image presentation was random, and the time required to assess was also recorded. The sequential test comprised an observer experiment conducted for 50 cases with and without the App. The observers first evaluated breast density without the App. Subsequently, the results of the App were presented immediately, and after viewing the results of the App, the observers evaluated the breast density again. The results of the App were displayed on a 5-megapixel high-definition monitor (JVCKENWOOD), and the room was darkened during the experiment, similar to a general reading environment. Both tests require training before the experiment to ensure that the criteria for evaluation are not altered [25]. The observers were instructed on the display and characteristics of the App before the experiment in this study and provided sufficient training to familiarize themselves with the display and performance of the App.

Creating datasets and truths

The dataset used in this study comprised mammograms (Pe･ru･ru, Canon Medical Systems Corporation, Tochigi, Japan) acquired between January 2020 and August 2020 at Otsuka Breastcare Clinic that was anonymously processed. The guideline-recommended medio-lateral-oblique (MLO) view of the mammograms was used in this study. Images of the dataset were evaluated by an expert breast radiologist with extensive experience, who is one of the main members of the QABCS guideline development team, to create the dataset for assessment. The mammary gland content ratio (0-100%) and breast density (1, fatty; 2, scattered; 3, heterogeneously dense; and 4, extremely dense) were evaluated. The expert evaluated the images subjectively and responded orally. The experimenter displayed the images and recorded the evaluations of the expert. If the left and right breasts showed different evaluation results, the result with the higher evaluation was adopted. The same subjective evaluation was used in both observer experiments. The training, independent, and sequential test datasets comprised different cases. Thirty cases (fatty, 4; scattered, 11; heterogeneously dense, 11; and extremely dense, 4) were used for training. A total of 100 cases (fatty, 5; scattered, 56; heterogeneously dense, 36; and extremely dense, 3) were used in the independent tests. Fifty cases (fatty, 3; scattered, 27; heterogeneously dense, 18; and extremely dense, 2) were used in the sequential test. The training, independent, and sequential datasets were established according to the Japanese breast density, with a high number of *scattered* and *heterogeneously dense* breasts and a low number of *fatty* and *extremely dense* breasts [26]. The experimental dataset was created according to the actual processing accuracy using the agreement rates between the expert’s evaluation and the results of the App. The agreement rates for all four and two categories of breast density were 72% and 83%, respectively. The agreement rates for the *fatty*, *scattered*, *heterogeneously dense*, and *extremely dense* categories were 9%, 79%, 74%, and 47%, respectively.

Method of assessments

The following three factors were considered while assessing the effectiveness of the App: (1) inter-observer variability, (2) expert-to-observer variability, and (3) the time required for the observer experiment. The results obtained with and without the use of the App were compared. (1) The standard deviation of the responses of the observers for mammary gland content ratio and breast density in each case was assessed to compare the inter-observer variability; a *t*-test was used to determine significant differences. The cases were divided according to the four categories of breast density for a detailed analysis, and each of the two categories (*fatty breast *and *dense breast*) was tested in the same manner. Intraclass correlation coefficients (ICC) of the mammary gland content ratio were also assessed. (2) The differences between the responses of the observers and the expert for the mammary gland content ratio were assessed using a *t*-test to determine the expert-to-observer variability. The agreement rate and Cohen's kappa (κ) between the responses of the observers and the expert for breast density were calculated. (3) The time required to complete the observer experiments was recorded to compare the time. For (2) and (3), the observers were also evaluated according to their years of experience (<5 years, 5-15 years, and ≥15 years). The independent tests assessed (1), (2), and (3), whereas the sequential tests assessed only (1) and (2). RStudio (version 1.1.456) was used to calculate ICCs and Cohen's kappa (κ).

## Results

Independent test

**Figure 3:**
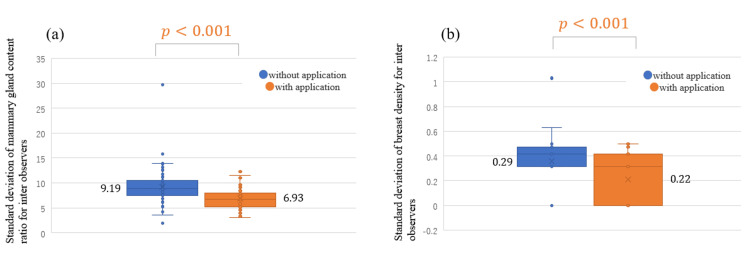
Standard deviation of observers’ responses for the (a) mammary gland content ratio and (b) breast density for all cases with and without the use of the App in the independent test.

Figure 3 presents the standard deviation of the observers' responses for (a) the mammary gland content ratio and (b) breast density for all cases. For the mammary gland content ratio, the standard deviation for all cases was significantly lower with the use of the App (the average standard deviation without the App, 9.19; with the App, 6.93; *P* < 0.001). The ICC (2,1) of the responses of the observers for the mammary gland content ratio for all cases was also higher with the use of the App (without, 0.737; with, 0.877). For breast density, the standard deviation for all cases was significantly lower with the use of the App (the average standard deviation without the App: 0.29, with the App, 0.22; *P* < 0.001). Category-specific analysis for the mammary gland content ratio and breast density revealed that the standard deviations were significantly lower with the use of the App in the following order: *scattered*, *heterogeneously dense*, *fatty breast*, and *dense breast* (Table 1).

**Table 1:** Standard deviation of observer responses for the mammary gland content ratio and breast density in the category-specific analysis with and without the use of the App in the independent test. SD, standard deviation

		Number of images	Mammary gland content ratio			Breast density		
			SD Average		*P*-value	SD Average		*P*-value
			Without	With		Without	With	
Four categories	Fatty	5	5.16	4.80	0.820	0.18	0.34	0.274
	Scattered	56	9.42	7.04	< 0.001	0.32	0.17	< 0.001
	Heterogeneously dense	36	8.96	7.13	< 0.001	0.41	0.25	< 0.001
	Extremely dense	3	14.40	6.07	0.425	0.62	0.30	0.455
Two categories	Fatty breast	61	9.07	6.86	< 0.001	0.31	0.18	< 0.001
	Dense breast	39	9.38	7.05	< 0.001	0.43	0.26	< 0.001

**Figure 4:**
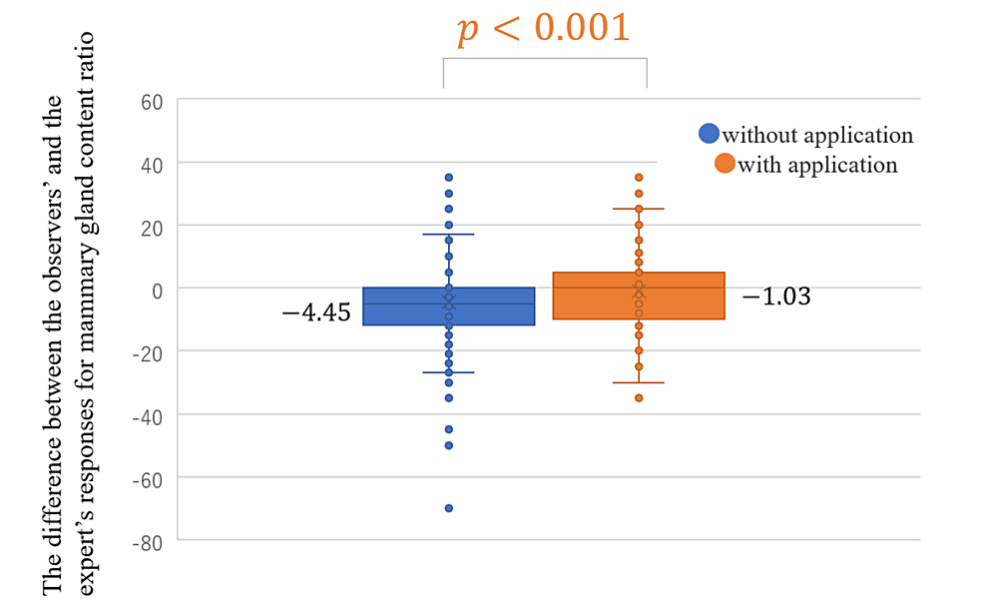
Difference between the responses of the observers and the expert for mammary gland content ratio with and without the use of the App in the independent test.

**Figure 5:**
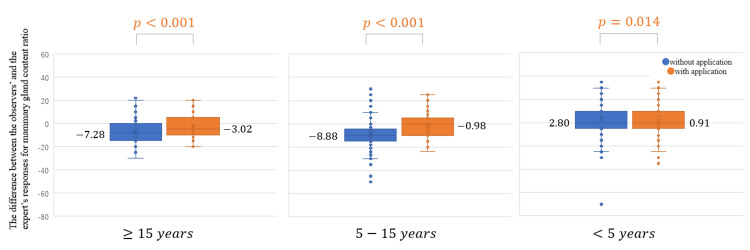
Difference between the responses of the observers and the expert for mammary gland content ratio according to the years of experience with and without the use of the application in the independent test.

The difference between the responses of the observers and the expert for mammary gland content ratio became significantly closer to zero with the use of the App (the average of the difference without the App, -4.45, and with the App, -1.03; *P* < 0.001; Figure 4). It was closer to the expert’s responses with the use of the App for all years of experience with statistical significance (Figure 5). The agreement rate between the responses of the observers and the expert for the four categories of breast density was improved by 6.6% with the use of the App: without the App, 72.2% (κ = 0.511) (Table 2) and with the App, 78.8% (κ = 0.626)(Table 3). The agreement rate between the responses of the observers and the expert in the analysis of two categories was improved by 4.8% with the use of the App: without the App 81.8% (κ = 0.604) (Table 4) and with the App 86.6% (κ = 0.723) (Table 5). The agreement rate between the observers’ and the experts’ responses for breast density evaluation improved for all years of experience with the use of the App: four categories (Table 6) and two categories (Table 7).

**Table 2:** Results of the comparison across all four categories of breast density according to the responses of the observers and the expert (without the App) in the independent test. Agreement rate：72.2%, kappa = 0.511.

Without the App		Result			
		Fatty	Scattered	Heterogeneously dense	Extremely dense
Truth	Fatty	39	6	0	0
	Scattered	55	398	51	0
	Heterogeneously dense	0	111	199	14
	Extremely dense	1	1	11	14

**Table 3:** Results of the comparison across all four categories of breast density according to the responses of the observers and the expert (with the App) in the independent test. Agreement rate: 78.8%, kappa = 0.626.

With the App		Result			
		Fatty	Scattered	Heterogeneously dense	Extremely dense
Truth	Fatty	31	14	0	0
	Scattered	16	409	79	0
	Heterogeneously dense	0	42	255	27
	Extremely dense	0	0	13	14

**Table 4:** Results of the comparison between two categories of breast density according to the responses of the observers and the expert (without the App) in the independent test. Agreement rate：81.8％, kappa = 0.604.

Without the App		Result	
		Fatty breast	Dense breast
Truth	Fatty breast	498	51
	Dense breast	113	238

**Table 5:** Results of the comparison between two categories of breast density according to the responses of the observers and the expert (with the App) in the independent test. Agreement rate: 86.6％, kappa = 0.723.

With the App		Result	
		Fatty breast	Dense breast
Truth	Fatty breast	470	79
	Dense breast	42	309

**Table 6:** Agreement rate between the responses of the observers and the expert for breast density according to the years of experience with and without the use of the App in the independent test (four categories). Agreement rate (%).

Four categories			
Years of experience	Without the App	With the App	Without - With
≥15 years	77.7	80.7	3.0
5-15 years	65.3	78.3	13.0
<5 years	73.7	77.3	3.7
Average	72.2	78.8	6.6

**Table 7:** Agreement rate between the responses of the observers and the expert for breast density according to the years of experience with and without the use of the App in the independent test (two categories). Agreement rate (%).

Two categories			
Years of experience	Without the App	With the App	Without - With
≥15 years	84.0	87.3	3.3
5-15 years	80.7	88.3	7.7
<5 years	80.7	85.7	5.0
Average	81.8	86.6	4.8

In terms of the time required without the App and with the App, the use of the App led to a reduction of 186.9 seconds (1.8 seconds per case) in the average time required by the nine observers to evaluate 100 cases (Table 8).

**Table 8:** Time required for the evaluation of breast density according to years of experience with and without the use of the App in the independent test Time required for evaluation (seconds)

Years of experience	Without the App	With the App	Without - With
≥15 years	756.3	664.3	92.0
5-15 years	1084.7	941.7	143.0
<5 years	1032.0	706.3	325.7
Average	957.7	770.8	186.9

Sequential test

**Figure 6:**
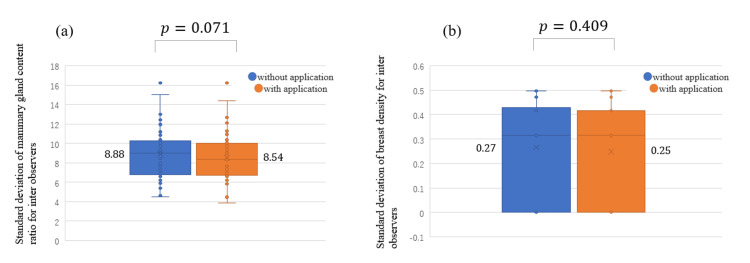
Standard deviation of observers' responses for the (a) mammary gland content ratio and (b) breast density for all cases with and without the use of the App in the sequential test.

Figure 6 shows the standard deviation of the responses of the observers for (a) the mammary gland content ratio and (b) breast density for all cases. For the mammary gland content ratio, the standard deviation for all cases was not significantly lower with the use of the App (the average of the standard deviation without the App, 8.88; with the App, 8.54; *P* = 0.071). The ICC (2,1) of the responses of the observers for mammary gland content ratio for all cases was slightly higher with the use of the App (without, 0.794; with, 0.807). For breast density, the standard deviation for all cases was not significantly smaller with the use of the App (the average of the standard deviation without the App, 0.27; with the App, 0.25; *P* = 0.409). The standard deviations were not significantly lower with the use of the App in this category-specific analysis of the mammary gland content ratio and breast density (Table 9).

**Table 9:** Standard deviation of the observer responses for the mammary gland content ratio and breast density in the category-specific analysis with and without the use of the App in the sequential test. SD, standard deviation

		Number of images	Mammary gland content ratio			Breast density		
			SD average		*P*-value	SD average		*P*-value
			Without	With		Without	With	
Four categories	Fatty	3	7.99	7.76	0.422	0.42	0.38	0.423
	Scattered	27	10.09	9.61	0.124	0.27	0.23	0.203
	Heterogeneously dense	18	7.38	7.27	0.668	0.21	0.24	0.328
	Extremely dense	2	7.54	6.77	0.200	0.48	0.42	0.117
Two categories	Fatty breast	30	9.88	9.42	0.106	0.28	0.24	0.166
	Dense breast	20	7.39	7.22	0.445	0.24	0.26	0.486

**Figure 7:**
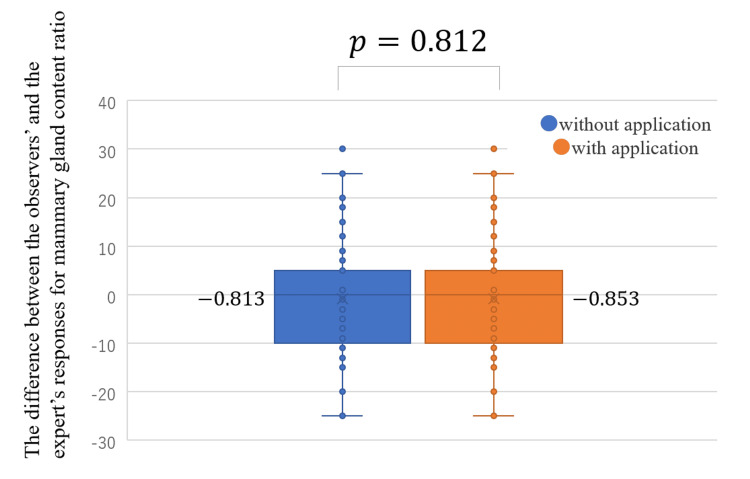
Differences between the responses of the observers and the expert for the mammary gland content ratio with and without the use of the App in the sequential test.

**Figure 8:**
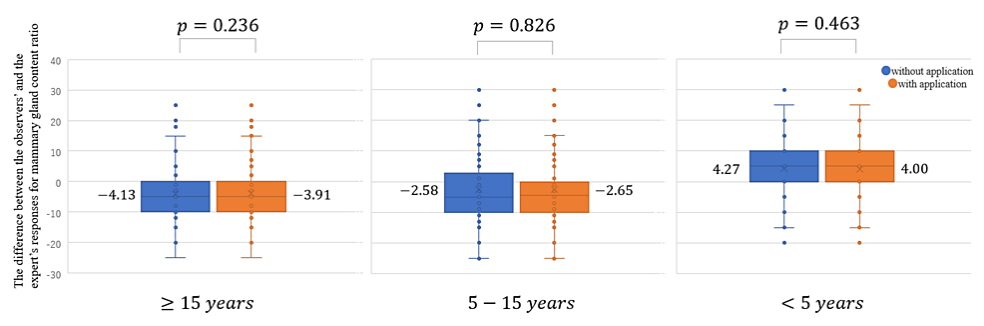
The difference between the responses of the observers and the expert for the mammary gland content ratio according to the years of experience with and without the use of the application in the sequential test.

The difference between the responses of the observers and the expert for the mammary gland content ratio did not become significantly closer to zero with the use of the App (the average of the difference without the App, -0.813; with the App, -0.853; *P* = 0.812; Figure 7). The use of the App did not result in a statistically significant reduction for all years of experience (Figure 8). The agreement rate between the responses of the observers and the expert for the four categories of breast density did not improve with the use of the App: without the App, 82.0% (κ = 0.687) (Table 10); with the App, 81.6% (κ = 0.679) (Table 11). Analysis using the two categories revealed that the agreement rate between the responses of the observers and the expert did not improve with the use of the App: without the App, 87.1% (κ = 0.734) (Table 12); with App, 86.9% (κ = 0.730) (Table 13). The use of the App improved the agreement rate between the observers’ and the expert’s responses for breast density evaluation only for those with 5-15 years of experience: four categories (Table 14) and two categories (Table 15). 

**Table 10:** Results of the comparison across all four categories of breast density according to the responses of the observers and the expert (without the App) in the sequential test. Agreement rate：82.0%, kappa = 0.687.

Without the App		Result			
		Fatty	Scattered	Heterogeneously dense	Extremely dense
Truth	Fatty	21	6	0	0
	Scattered	7	202	34	0
	Heterogeneously dense	0	24	135	3
	Extremely dense	0	0	7	11

**Table 11:** Results of the comparison across all four categories of breast density according to the responses of the observers and the expert (with the App) in the sequential test. Agreement rate：81.6%, kappa = 0.679.

With the App		Result			
		Fatty	Scattered	Heterogeneously dense	Extremely dense
Truth	Fatty	22	5	0	0
	Scattered	4	204	35	0
	Heterogeneously dense	0	24	132	6
	Extremely dense	0	0	9	9

**Table 12:** Results of the comparison between two categories of breast density according to the responses of the observers and the expert (without the App) in the sequential test. Agreement rate：87.1%, kappa = 0.734.

Without the App		Result	
		Fatty breast	Dense breast
Truth	Fatty breast	236	34
	Dense breast	24	156

**Table 13:** Results of the comparison between two categories of breast density according to the responses of the observers and the expert (with the App) in the sequential test. Agreement rate：86.9%, kappa = 0.730.

With App		Result	
		Fatty breast	Dense breast
Truth	Fatty breast	235	35
	Dense breast	24	156

**Table 14:** Agreement rate between the responses of the observers and the expert for breast density according to years of experience with and without the use of the App in the sequential test (four categories). Agreement rate (%).

Four categories			
Years of experience	Without the App	With the App	Without - With
≥15 years	88.0	86.7	-1.3
5-15 years	80.7	84.0	3.3
<5 years	77.3	74.0	-3.3
Average	82.0	81.6	-0.4

**Table 15:** Agreement rate between the responses of the observers and the expert for breast density according to years of experience with and without the use of the App in the sequential test (two categories). Agreement rate (%).

Two categories			
Years of experience	Without the App	With the App	Without - With
≥15 years	92.0	89.3	-2.7
5-15 years	86.7	88.7	2.0
<5 years	82.7	82.0	-0.7
Average	87.1	86.9	-0.2

## Discussion

In the present study, the independent test results indicated that the use of the App decreased the variation in the mammary gland content ratio and breast density among breast radiologists. The categorical analysis revealed a reduction in the *scatter* and *heterogeneously dense* categories in the four-category analysis and *fatty* and *dense* variation in the two-category analysis by the App. The reduction in inter-radiologist variability in the *scatter* and *heterogeneously dense* categories, which have historically low inter-radiologist agreement, suggests that using the App could mitigate the inter-radiologist differences in breast density notifications. Similarly, using the App reduced the variation in the mammary gland content ratio and breast density compared to the evaluations by the experts. This trend was consistent among all breast radiologists with varying years of experience. In particular, the agreement rate improved for the *scattered* and *heterogeneously dense* categories, where errors in evaluation are considered fatal for all four categories of breast density. Extremely dense cases were incorrectly categorized as *fatty* or *scattered*, which represent miscategorization to categories that are three and two steps away, respectively, without the use of the App, suggesting inadvertent errors. However, miscategorization by two or more steps was no longer observed with the use of the App. The agreement rate for two categories of breast density improved for *dense breasts*, which is common among Japanese women. Thus, the use of the App is presumed to have mitigated errors, such as inadvertent errors, while supporting the evaluation of important categories. Maintaining the accuracy of the evaluation by the breast radiologists with the use of the App is paramount. Notably, the App also yielded satisfactory results in this respect. In addition, the App shortened the evaluation time. Given the substantial volume of images involved in mammograms for breast cancer screening, a shortened evaluation time can alleviate the burden on breast radiologists. Moreover, the time required for evaluation was reduced for all breast radiologists with varying years of experience, with the rate of reduction being greater for younger breast radiologists. Thus, the use of the App by younger breast radiologists is expected to significantly improve their workflow. The App is also effective as an educational tool for younger breast radiologists, as it reduces the variability in evaluation between experienced and younger breast radiologists and significantly reduces the time required for evaluation.

In contrast, the results of the sequential test did not yield any results that could suggest the effectiveness of the App. Table 16 shows the number and percentage of participants who changed their evaluations after using the App in the sequential test. The percentage of cases that changed their evaluations resulted in an average of less than 10% of the cases. Psychological factors, such as difficulty in revising the evaluations immediately after evaluating oneself, may have played a role. Or perhaps the reluctance to revise an evaluation based on the results of the App. The independent test substantially eliminates this factor, assuming that the one-month interlude removes any learning-effect bias. As shown in Table 16, the low percentage of patients with changes in their evaluation may also explain why no statistically significant difference was observed in the sequential test.

**Table 16:** Number and percentage of cases with changes in their evaluation after the use of the App in the sequential test.

Years of experience	Number of images, *n*	Proportion (%)
≥15 years	2	4.0
5-15 years	3.7	7.3
<5 years	6.3	12.7
Average	3.8	7.6

The results of the independent and sequential tests suggest that the usefulness of the App can be maximized by its concurrent use, which can alleviate the variability in breast density evaluations among breast radiologists and shorten the breast density evaluation time without compromising the accuracy of the evaluation.

Ultrasonography, which renders normal breast tissues bright and abnormal lesions dark, is effective for screening *dense breasts*, which are common among Asian women, including Japanese women. However, no data are demonstrating the effectiveness of combined mammography and ultrasonography in reducing breast cancer mortality. The J-START research team is conducting a prospective study in Japan to demonstrate the effect of combined mammography and ultrasonography for *dense breasts* in reducing breast cancer mortality [27,28]. Given this evolving landscape, there is a growing trend toward recommending the combined use of mammography and ultrasonography for *dense breasts*, and it is likely that the concept of *dense breasts* will continue to become more important for breast cancer screening in the future.

Two limitations of this study are discussed as follows. In the present study, a questionnaire was employed to confirm whether learning-effect bias was avoided. All breast radiologists responded that they did not perceive the cases without and with the App as identical or that they noticed that some of the characteristic cases were memorable but had no effect on the evaluation of breast density. These results suggest that the bias of the learning effect was avoided. Nevertheless, the possibility that the increase in efficiency due to familiarity with the task led to a reduction in the evaluation time must be considered. Although the datasets were matched to the proportion of breast density in Japan, there remains a large bias that needs to be examined in the future.

## Conclusions

Independent and sequential tests were conducted with nine breast radiologists to determine the usefulness of the App by evaluating the mammary gland content ratio and breast density. The concurrent-look mode of the App reduced the inter-observer and expert-to-observer variability, which also led to a reduction in the evaluation time. The findings of this study suggest that the use of the App for breast density analysis improved reading efficiency.
